# Recent advances in imaging and understanding interstitial cystitis

**DOI:** 10.12688/f1000research.16096.1

**Published:** 2018-11-09

**Authors:** Pradeep Tyagi, Chan-Hong Moon, Joseph Janicki, Jonathan Kaufman, Michael Chancellor, Naoki Yoshimura, Christopher Chermansky

**Affiliations:** 1 Urology, University of Pittsburgh Medical Center, Pittsburgh, Pennsylvania, 15213, USA; 2 Radiology, University of Pittsburgh Medical Center, Pittsburgh, Pennsylvania, 15213, USA; 3 Lipella, Pittsburgh, USA; 4 Urology, OU- Beaumont school of medicine, Royal oak, USA

**Keywords:** Interstitial cystitis, bladder wall, imaging, MRI, radiation, fibrosis

## Abstract

Interstitial cystitis/bladder pain syndrome (IC/BPS) is a debilitating condition associated with intense pelvic pain and bladder storage symptoms. Since diagnosis is difficult, prevalence estimates vary with the methodology used. There is also a lack of proven imaging tools and biomarkers to assist in differentiation of IC/BPS from other urinary disorders (overactive bladder, vulvodynia, endometriosis, and prostatitis). Current uncertainty regarding the etiology and pathology of IC/BPS ultimately impacts its timely and successful treatment, as well as hampers future drug development. This review will cover recent developments in imaging methods, such as magnetic resonance imaging, that advance the understanding of IC/BPS and guide drug development.

## Introduction

Interstitial cystitis/bladder pain syndrome (IC/BPS) is a debilitating pelvic pain condition associated with bladder storage symptoms. The International Continence Society (ICS) and the European Society for the Study of IC/BPS (ESSIC) define IC/BPS as a condition with chronic (>6 months) pelvic pain, pressure, or discomfort perceived to be related to the urinary bladder accompanied by at least one other urinary symptom like persistent urge to void or frequency with or without cystoscopic abnormalities. These hard-to-treat subjective symptoms often overlap with a host of other urinary disorders including urinary tract infection (UTI) ^1^, chronic urethral syndrome, overactive bladder, hypersensitive bladder, vulvodynia, endometriosis in women, and prostatitis in men. The RAND Interstitial Cystitis Epidemiology survey estimated that 3 to 8 million women and 1 to 4 million men in the United States may have symptoms consistent with the diagnosis of IC/BPS ^2^.

The variability of definitions (ICS, American Urological Association, ESSIC) and the abundance of non-specific symptoms and comorbidities complicate the diagnosis and management of IC/BPS ^3– 5^. Currently, the diagnosis of IC/BPS is typically based on the physician’s subjective assessment and exclusion of other conditions with overlapping symptoms ^6^, including UTI. Nevertheless, two distinct patient groups can be identified corresponding to patients with organic disease of the bladder wall ^7– 9^ and patients with painful hypersensitivity ^10^, experiencing pain in the pelvic floor as well as other organs ^11, 12^. The ICS also recently updated the IC/BPS definition and proposed three distinct entities: pelvic hypersensitivity, IC/BPS, and IC/BPS with Hunner's lesions ^2^. But no objective criteria are available to differentiate patients with IC/BPS into separate entities as outlined by the updated the ICS’ definition.

Current uncertainty regarding the etiology and pathology of IC/BPS ultimately impacts its treatment and hampers future drug development. Similar situations in other organs, where a disease exhibit overlapping symptoms, have been elucidated by applying imaging methods and biomarkers. Following sections will cover the current understanding of the pathophysiology of IC/BPS and the potential of different imaging techniques, particularly magnetic resonance imaging (MRI) of the bladder, pelvic floor muscles, and brain, in advancing the understanding of IC/BPS.

## Current understanding of the pathology

### Interstitial cystitis/bladder pain syndrome originating from the bladder

IC/BPS patients of this category ^8, 9^ are considered to have an organic disease of the bladder wall. Pathological examination of biopsy specimens from the bladder wall of such IC/BPS patients typically finds a higher incidence of mucosal hemorrhages (glomerulations) submucosal inflammation ^13^ and a high density of mast cells ^14, 15^, which is associated with hyperexcitability ^16^ of afferent fibers ^17^. Cystoscopic detection of glomerulations in the bladder mucosa of IC/BPS patients is the defining characteristic of the ESSIC type 2C classification, while the detection of Hunner’s lesion (a denudation of urothelium) is a sign of classic type or ESSIC type 3C, more commonly referred to as Hunner-type IC/BPS. Hunner-type IC/BPS is seen in 5–10% of cases and the analysis of their bladder wall biopsy is generally associated with high expression of T and B cell markers ^18^, low expression of urothelial markers, high density of mast cells, focal lymphoid aggregates, overexpression of IL-6, IL-10, IL-17A, and inducible NOS (iNOS) mRNA ^14^. Analysis of urine specimens found higher concentration of immunoglobulin and inflammatory mediators. Our current understanding is that most IC/BPS patients in the population do not present with Hunner’s lesions and therefore are classed as having non-Hunner-type IC/BPS ^19^. To date, bladder wall biopsy is the only available method to detect mast cells ^20^ and molecular signatures of chronic inflammation in the bladder wall, but the method is limited by invasiveness and potential complications. Different fixation/staining techniques for mast cells ^21^ also add to the confusion. Therefore, there is a need for a non-invasive imaging method to identify patients with organic disease of the bladder wall.

### Contribution of bladder permeability and interstitial cystitis/bladder pain syndrome

Multiple lines of evidence now support a role for increased bladder permeability in IC/BPS patients with organic disease of the bladder wall ^22– 24^. Denudation of the glycosaminoglycan (GAG) layer located at the luminal surface of the bladder ^24, 25^ by protamine sulfate leads to increased bladder permeability, which can be ameliorated by long-term oral or intravesical administration of GAG substitutes. Past published studies used the serum uptake of intravesically instilled urea ^24^ or radionuclide ^26^ as an index of bladder permeability. However, serum uptake ^26^ of instilled substances is subject to considerable inter-individual variability in the distended bladder wall thickness ^27^ and the volume of systemic distribution. Large divergence in the published findings ^24, 26^ can easily be explained by the differences in the molecular weight (60 versus 487 Da) of urea and the radionuclide used as a probe.

The contribution of bladder permeability to the pathophysiology of IC/BPS is also supported by the immunohistochemical and ultrastructural studies of biopsies taken from IC/BPS patients. Decreased expression of tight junction protein and adhesive junction proteins including E-cadherin and zonula occludens-1 (ZO-1) as well as the differentiation marker uroplakin ^22, 28^ supports the role of bladder permeability in IC/BPS pathogenesis. Electron microscopy of the urothelium in the biopsies revealed the contribution of leaky tight junctions in IC/BPS ^23^. Umbrella cells are pleomorphic in the apical layer of the urothelium, and a decrease in the microplicae (ridges) in umbrella cell membranes was suggested to be pathognomonic for IC/BPS ^29^. Furthermore, upregulation of the purinoceptor P2X3 and an increased release of adenosine triphosphate from the urothelium are also reported in IC/BPS patients ^30^. However, biopsy-based studies are sensitive to site selection, which can lead to the apparent variability in the morphological differences between the urothelium of IC/BPS patients and controls ^29, 31, 32^.

### Contribution of fibrosis in interstitial cystitis/bladder pain syndrome

**Figure 1.**
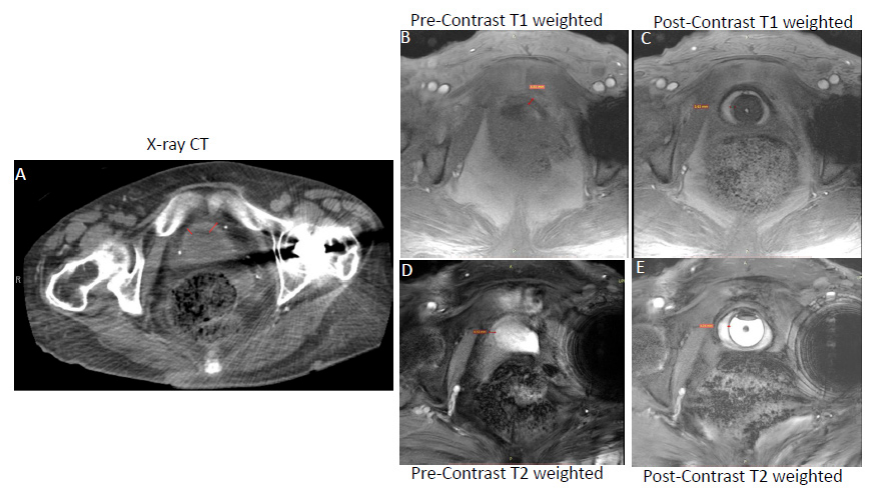
Magnetic resonance imaging (MRI) is superior to computed tomography (CT) in bladder wall segmentation. Contrast-enhanced T _1_-weighted MRI (Panel **C**) is superior to CT (Panel **A**) and unenhanced T _1_-weighted MRI (Panel **B**) for resolving the thickened bladder wall of the same female ulcerative interstitial cystitis/bladder pain syndrome (IC/BPS) patient as indicated by a red line drawn across the bladder wall. Representative T _1_-weighted fast low angle shot (FLASH) images acquired at the flip angle of 14° in orthogonal orientation before (Panel **B**) and after novel contrast mixture (NCM) instillation (Panel **C**) demonstrate that NCM-enhanced MRI can non-invasively segment the bladder wall into a middle layer of bright signal intensity sandwiched between inner and outer layers of lower signal intensity. Fibrotic changes reported in the pathological results of the biopsy from the IC/BPS patient are visible as the bright middle layer from the diffusion of gadobutrol from NCM instilled in the lumen (hypointense circular region in Panel **C**). Fast acquisition of a single slice (5 mm thickness) over a single breath hold of approximately 17 seconds with imaging parameters of repetition time (TR)/echo time (TE) of 5.5/2 milliseconds, field of view of 180×180 mm ^2^, matrix of 256×256 and 10 averaging achieved in-plane resolution of 0.35 mm (after 2x interpolation) with minimal influence of motion and chemical shift artifacts (Panel **B** and **C**). Akin to CT (Panel **A**), T _1_-weighted MRI (Panel **B**) with similar imaging parameters as in Panel **C** could not resolve the bladder wall from the lumen in the absence of NCM owing to the relatively long intrinsic T _1_ relaxation time of >1 second for the bladder wall and of urine in the lumen. Although the bladder wall is demarcated clearly in pre-contrast T _2_-weighted image (Panel **D** and **E**) acquired at the TR/TE of 6.9/3.1 milliseconds, there is underestimation of bladder wall thickness, especially of the inner layer from 3.62 mm in panel C to 1.21 mm in Panel **E**. Instillation of gadobutrol in the absence of ferumoxytol enhances the T _1_ contrast of urine as well as the bladder wall, which precludes it from affording improved image contrast. This figure is an original image taken in our clinic for this publication.

Fibrosis, or tissue scarring, often results from the evolutionarily conserved process of wound healing to resolve chronic inflammation. Progressive fibrotic changes in the bladder wall of IC/BPS patients are characterized by excessive deposition of extracellular matrix within the lamina propria and smooth muscle ( Figure 1), generation of contractile fibroblasts (myofibroblasts), and decreased capillary density ^33, 34^. These histological changes are associated with the upregulation of collagen genes, collagen I, collagen III, fibronectin, and transforming growth factor-β1 (TGF-β1) ^35^ and the downregulation of sonic hedgehog, WNT gene family, *WNT2B*, *WNT5A*, *WNT10A*, and *WNT11* in the biopsy of non-Hunner-type IC/BPS patients ^33, 36– 40^. Recent studies report the association of YKL-40 antigenic expression in detrusor mast cell granules and submucosal macrophages with detrusor fibrosis ^34^ and of the fibrosis in ketamine-induced ^41^ IC/BPS with the activation of mammalian target of rapamycin (mTOR).

Changes in collagen gene expression in biopsy specimens representing the mucosa and submucosa tissue layers of the bladder wall are consistent with the increased collagen staining in the intra- and inter-fascicular muscle tissue of IC/BPS patients ^15^. Histological detection of fibrosis in the bladder wall is associated with increased passive tissue stiffness with stretch, reduced bladder compliance ^42^, uninhibited detrusor contractions ^43^, higher urinary frequency, and lower bladder capacity ^43^. However, decreased bladder capacity ^7^ is not a specific marker for IC/BPS patients, as both IC/BPS and overactive bladder patients ^44^ exhibit decreased bladder capacity. Since fibrotic changes in non-Hunner-type IC and Hunner-type IC ^15, 33, 34, 45^ are significantly associated with the failure of standard treatment and the need for aggressive treatment options of steroids or hydrodistension with deep bladder biopsies, pain clinic, or surgery (e.g. urinary diversion and cystectomy) in extreme cases. Therefore, earlier detection of fibrotic changes can guide the pre-emptive addition of anti-fibrotic therapy for improved treatment outcomes.

## Imaging techniques

Average thickness of human bladder wall is approximately 3 mm and is composed of detrusor smooth muscle sandwiched between the mucosa and the adventitia ^46^, which makes it challenging to image by available planar and tomographic imaging techniques.

### Planar imaging techniques

***Cystoscopy***. Cystoscopy is a planar imaging technique with a small field of view (FOV) for direct, real-time image guidance of the bladder wall. The ICS regards cystoscopy as the standard technique to identify IC/BPS patients with Hunner’s lesions ^47^. Two-dimensional (2D) images generated via cystoscope show Hunner’s lesions on the luminal surface of bladder wall as either singular or multiple erythematous mucosal patches, with small vessels radiating toward a discrete central pale scar with fibrin or coagulum. Fissure and glomerulations (petechial hemorrhages) are visible during bladder emptying after hydrodistention with cascade bleeding. The absence of Hunner’s lesions on cystoscopic examination classifies the patients as non-Hunner-type IC/BPS. The detection of Hunner’s lesions can guide appropriate surgical treatment of the lesion including transurethral cauterization or fulguration and consideration of hydrodistension under general anesthesia in the absence of Hunner’s lesions ^48^. However, small FOV of cystoscopy limits its sampling to small tissue volumes, and it can easily miss bladder wall changes that are morphologically indistinct from normal bladder wall ^49^. Moreover, this gross morphological discrimination is unable to exclude patients with an abnormality in their pelvic floor.

***Ultrasound***. Available ultrasound techniques to image the bladder wall produce widely variable outcomes depending on the anatomical approach (translabial/transperineal and suprapubic), frequency, and other factors ^46^. Detrusor smooth muscle is hypoechogenic (dark), and the mucosa and the outer layer of the adventitia are hyperechogenic (bright). Since bladder wall and detrusor become thinner as the bladder fills, values of the bladder wall thickness (BWT) drop by about 1 mm for each increase in bladder volume distension by 50 mL. Therefore, most measurements of BWT in the range of 3 mm to 6.5 mm are made with bladder filling volume of less than 50 mL using a 5–9 MHz probe. Since image resolution with ultrasound is only limited to 1 mm, a distended bladder wall may only be represented by around 3 image pixels. Ultrasound is generally used to detect renal involvement, pelvic floor muscle mobility ^50^, and thickening of the bladder wall in recurrent UTI ^51, 52^ and in IC/BPS ^53^ patients.

**Figure 2.**
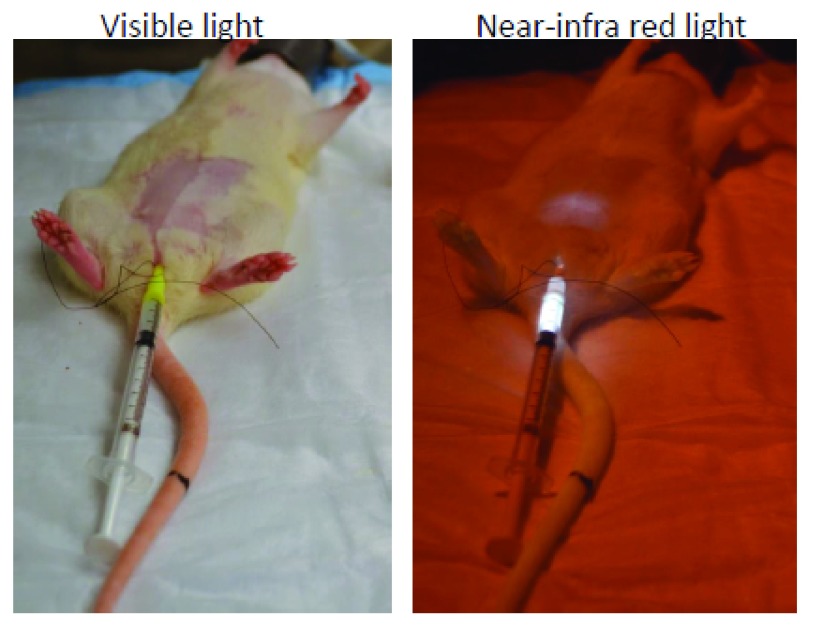
Near infra-red imaging. Representative images for the rat pelvic area in visible light and in near infra-red (NIR) light after instillation of liposomes containing a trace amount of NIR dye. Deep tissue imaging of the bladder wall is possible, as light emitted from the bladder lumen in the NIR band encounters low background auto-fluorescence and minimal absorption by tissue components. This figure is an original image taken in our clinic for this publication.

***Near-infrared imaging***. The application of conventional fluorescent microscopy for imaging the bladder wall is limited owing to the issues pertaining to absorption by tissues, scattering, and auto-fluorescence of visible light ^54^. Recently, fluorochromes emitting in the near-infrared (NIR) band were shown to emit light with tissue penetration approaching 10–15 cm ^55^. Low background auto-fluorescence and minimal absorption by tissue components ^54^ in the NIR band make it suitable for deep-tissue imaging ^56^ ( Figure 2). In a rodent study, the instillation of liposomes containing NIR dye allowed visualization of the instilled liposomes in anaesthetized mouse bladder wall ^57^. NIR spectroscopy (NIRS) is a non-invasive, functional, transcutaneous optical technique that uses NIR light to monitor changes in the concentration of oxyhemoglobin and deoxyhemoglobin in the bladder wall. Thus, optical monitoring of bladder wall oxygenation is feasible with NIRS ^58^ to potentially delineate the contribution of ischemia to IC/BPS symptoms.

### Tomographic imaging techniques

Tomographic imaging techniques are capable of three-dimensional (3D) display and acquisition of multiple slices across multiple planes. Such a large image volume is enormously rich in potential information, but the complex instrumentation and intensive image analysis preclude real-time feedback. The potential of tomographic image visualization and quantitative analysis in the diagnosis and treatment of IC/BPS remains unrealized.

***X-ray computed tomography***. Abdominal computed tomography (CT) is a tomographic imaging technique which was recently used to detect the treatment-associated decrease in bladder wall thickening of IC/BPS patients ^59^, identify hidden lesions under scanned area, and exclude malignancies. However, CT exposes patients to high doses of ionizing radiation (X-ray) and lacks the sensitivity to detect the onset and progression of fibrosis. Figure 1 illustrates the relative contrast resolution (degree of difference between brightest and darkest component of an image) of the bladder wall with CT and MRI.

***Magnetic resonance imaging***. MRI is a safe imaging technique that does not rely on ionizing radiation for tomographic imaging of visceral organs. MRI affords higher contrast and spatial resolution of soft tissue in multiplanar images ^60^, which makes it one of the preferred imaging modalities in urology. MRI is especially superior to CT in reliably producing slices more suited to detect lesions in the dome and base of the bladder wall. MRI also allows diffusion imaging and dynamic contrast enhancement in the bladder wall ^60^. However, there is no reference signal for MRI corresponding to the standard reference signal of 0 Hounsfield units for the radio density of water in CT. So, MR image quality in terms of both image contrast ( Figure 1) and spatial resolution is described by contrast resolution or contrast-detail measured by contrast–noise ratio (CNR) ^61^.

**Conventional MRI**

Exquisite soft tissue contrast in conventional T _1_- and T _2_-weighted MRI arises principally from the differences in the intrinsic tissue relaxation times T _1_ (spin–lattice relaxation time) ^62^ and T _2_ (spin–spin relaxation time) ^10, 63, 64^. Variation in signal sensitivity (rise in CNR) is to highlight the differences in T _1_^62^ and T _2_^63, 64^ for T _1_-weighted and T _2_-weighted images ^63, 65^ is commonly achieved by manipulating the acquisition parameters (e.g. flip angle [FA], echo time [TE], repetition time [TR], inversion time, etc.). Unenhanced T _1_-weighted MRI requires a TR of >4–5 times the T _1_ relaxation time of the target organ. Hence, a relatively long intrinsic T _1_ relaxation time of >1 second for bladder wall and of >6 seconds for urine at magnetic field strength of 3 Tesla (T) ^62, 66^ requires a TR of >5 seconds, which prolongs the image acquisition (>5 minutes) ^67^ and the resulting image quality of the thin human bladder wall ^68, 69^ becomes highly susceptible to artifacts from involuntary bowel and respiratory motion of the subject ^70^. Moreover, multiple signal averages ^63^ with prolonged image acquisition for T _1_-weighted MRI of the bladder wall can achieve in-plane resolution of >0.5 mm ^71, 72^.

We recently showed that the use of a short TR of 5.5 milliseconds increases the image resolution in T _1_-weighted MRI ( Figure 1B), but the isointense signal in the bladder wall and urine ^62^ makes it difficult to differentiate the thin human bladder wall from the urine. Though the human bladder wall is more distinguishable in T _2_-weighted MRI ( Figure 1D) ^63, 73^, T _1_-weighted MRI ^67^ is preferable for its higher signal-to-noise ratio (SNR) and accurate measurement of BWT ^69, 73– 75^ for characterizing bladder disorders ^51^. As shown in Figure 1, the thickness of the inner layer (urothelium) of the bladder wall is underestimated in T _2_-weighted images ^75, 76^. The bladder wall appears as a thin layer of intermediate intensity on T _2_-weighted images and is likely to correspond to the thickness indicated by of the middle and outer layer on the contrast-enhanced T _1_-weighted image ( Figure 1C) ^65^. It appears that the highly intense signal of urine in unenhanced T _2_-weighted images submerges the signal from a portion of the urothelium layer, leading to BWT underestimation ^75^. Since lesions in the bladder wall associated with IC/BPS are generally restricted to the urothelium, T _2_-weighted MRI can easily miss the important details for the pathological characterization of IC/BPS. T _2_-weighted MRI is also not suitable for staging the bladder cancer before invasion of muscular layer ^65^.

Furthermore, the sandwich composition of the bladder wall’s histology can displace the respective MR signals in the readout direction to produce chemical shift mis-registration of the perivesical fat signal and exaggeration of motion artifacts ^66, 77, 78^. Chemical shift artifact refers to the signal alterations that result from the 440 Hz differences in the resonant frequencies of fat and water protons at 3T ^66, 78^. Therefore, variable shape, location, and histological composition of the bladder wall limits the utility of conventional MRI in the diagnosis and treatment of IC/BPS. Nevertheless, urinary bladder can be visualized by MRI in multiple cross-sectional orientations with parallel image series in cross-sectional imaging planes and intersectional at different orientations. However, the information in 2D image slices is non-continuous owing to slice gaps. Hence, improvements in bladder wall imaging call for enhancement of contrast and SNR and reduced susceptibility to artifacts ^79^ during image acquisition and 3D reconstruction.

**Contrast-enhanced MRI**

One of the traditional approaches to enhance T _1_ contrast of the human bladder wall is the intravenous injection of gadolinium-based contrast agents (GBCAs). Entry of blood containing the injected GBCAs into the middle layer of the bladder wall can briefly differentiate it into three layers: a thin inner layer of low intensity, a middle layer of strong intensity, and a thick outer layer of intermediate intensity in post-contrast images ^65^. However, subsequent entry of the GBCA excreted into urine within 3 minutes of injection ^80, 81^, and subsequent continuous accumulation in bladder ultimately reduces the contrast between the lumen and bladder wall ^69, 73, 82^. Moreover, the T _2_ relaxation effect of GBCAs ^80, 83^ causes pseudolayering in the urine collected in the lumen ^73^. To overcome the drawbacks associated with the intravenous administration of GBCAs, several groups have now tried intravesical administration of GBCAs in bladder cancer ^84^, vesicoureteral reflux ^85^, and IC/BPS. But the enhancement of T _1_ contrast in the urine by the instilled GBCA analog gadolinium–diethylenetriaminepentaacetic acid prevents it from offering a good image contrast in the bladder wall ^69, 84, 86^ and accurately measuring the BWT. Likewise, instillation of superparamagnetic iron oxide (SPIO) nanoparticles ^87^ has also been tried without much success to improve the contrast of the human bladder wall ^88^.

**Figure 3.**
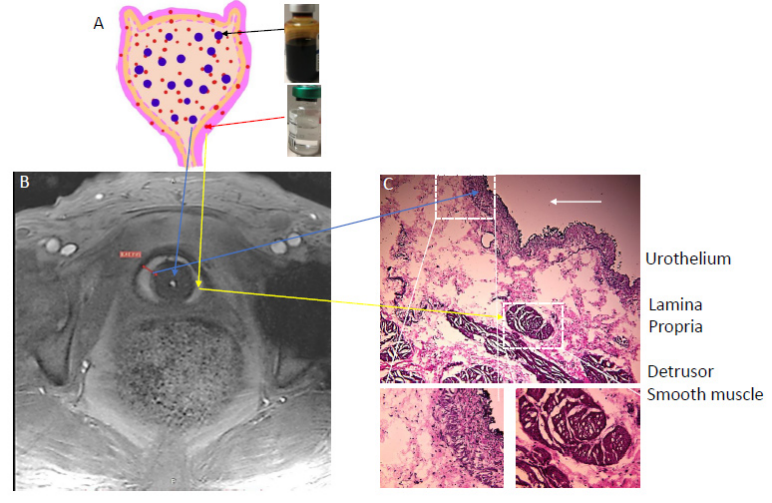
Contrast-enhanced magnetic resonance imaging (MRI) relies on the diffusion of gadolinium into the bladder wall. Novel contrast mixture-enhanced MRI relies on the differences in the contrast mechanisms and molecular weight of two US Food and Drug Administration-approved agents (gadobutrol diluted 1:250 and ferumoxytol diluted 1:104) for increasing the contrast resolution of the bladder wall (Panel **A**). Gadobutrol, a transparent liquid, is a gadolinium-based contrast agent (GBCA) with a molecular weight of 604.71 Da that reaches the extracellular space in the lamina propria to shorten T _1_ (positive contrast) or produce higher signal intensity (Panel **A** and **B**). Meanwhile, the large molecular weight of 750 kDa for ferumoxytol (vial with brown-colored liquid) restricts its bladder wall diffusion, and it produces a localized increase in proton dephasing, which decreases the signal intensity (negative contrast) in T _1_-weighted images. Instillation of novel contrast mixture 50 mL can non-invasively segment the inner layer (urothelium) and the outer layer of adventitia (dark signal) from the bright signal in the middle layer composed of the lamina propria and detrusor smooth muscle (Panel **C**). Bladder wall histology in Panel **C** is shown for illustration and does not represent the pathological characterization of the interstitial cystitis/bladder pain syndrome (IC/BPS) patient shown in Panel **B**. This figure is an original image taken in our clinic for this publication.

**Figure 4.**
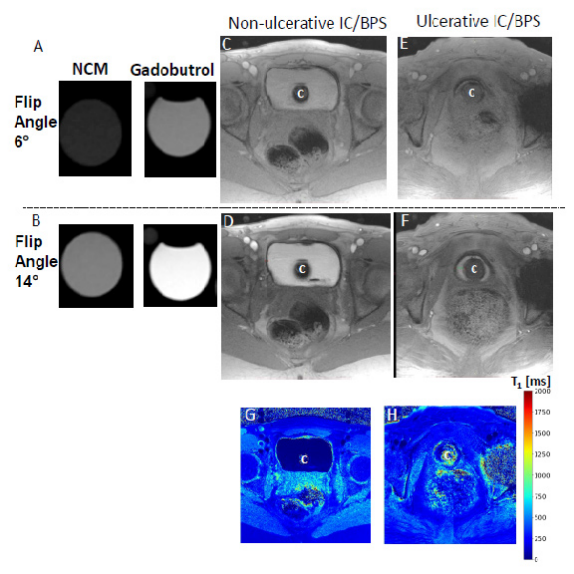
Quantitative measurement of gadolinium diffusion. T _1_-weighted fast low angle shot (FLASH) images with constant repetition time (TR) of 5.5 milliseconds (ms) at flip angle (FA) of 6° (Panel **A**, **C**, and **E**) and 14° (Panel **B**, **D**, and **E**) demonstrate that gadobutrol-mediated signal enhancement (visible in the right tube containing gadobutrol 4 mM alone) is suppressed by the presence of ferumoxytol (5 mM) in the novel contrast mixture (NCM) tube, as the gadobutrol concentration of 4 mM is the same in both tubes (Panel **A** and **B**). T _1_-weighted FLASH images demonstrates that greater separation of gadobutrol into the bladder wall away from the NCM instilled in the bladder lumen occurs in ulcerative IC/BPS patients (Panel **E** and **F**) than in non-ulcerative interstitial cystitis/bladder pain syndrome (IC/BPS) patient (Panel **C** and **D**), which is evident from the dramatic increase in signal intensity in Panel **F** relative to Panel **D** at FA of 14°. Catheter used for instillation is shown by **C** in panel **C**– **H**. Constant TR of 5.5 ms at different FAs achieves the stable steady-state conditions necessary for the differences in signal intensity of the same slice to become a function of T _1_ relaxation time as indicated by the color panel in Panel **H**. Greater shortening of T _1_ relaxation time (blue color) in ulcerative IC/BPS patients is consistent with higher diffusion of gadobutrol into the expanded extracellular matrix of the thickened bladder wall of IC/BPS patients. This figure is an original image taken in our clinic for this publication.

Our group recently reported that novel contrast mixture (NCM) can improve the image contrast of the bladder wall in T _1_-weighted MRI of rat ^88^ and human bladder ^61^ ( Figure 1C, Figure 3, and Figure 4). NCM is a homogenous mixture of gadobutrol (GBCA) diluted 1:250 and ferumoxytol (SPIO) diluted 1:104 in sterile water for irrigation (Panel A). Gadobutrol (transparent liquid) with a molecular weight of 604.71 Da, whether injected intravenously or instilled into bladder, reaches the extracellular space in the lamina propria to produce T _1_ contrast (signal increase). Meanwhile, the large molecular weight of 750 kDa restricts the bladder wall diffusion of ferumoxytol (vial with brown-colored liquid) to primarily exert T _2_ contrast (signal decrease) in the lumen and produce a localized increase in proton dephasing of T _1_-weighted FLASH (fast low angle shot) MRI at a TR/TE of 5.5/2 milliseconds. T _1_-weighted FLASH images of the tubes containing gadobutrol or NCM in Figure 4 A–B illustrate that the presence of ferumoxytol (5 mM) in the NCM tube masks the gadobutrol-mediated signal enhancement, which is visible with an increase in FA from 6° (panel A) to 14° (panel B) in the right tube containing gadobutrol 4 mM alone. The concentration of gadobutrol (4 mM) is the same in both tubes identified as NCM and gadobutrol. Likewise, the separation of gadobutrol away from NCM instilled in the lumen dramatically increases the signal intensity in the bladder wall of ulcerative IC/BPS patients ( Figure 4E–F) but not in the non-ulcerative IC/BPS patient ( Figure 4C–D), which indicates that a greater physical separation of gadobutrol from ferumoxytol occurs from the diffusion of gadobutrol into the bladder wall away from the NCM instilled in the bladder lumen of ulcerative IC/BPS patients.

Post-contrast images of human bladder wall were acquired after 50 mL NCM instillation to achieve artifact-free visualization of the bladder wall with an in-plane resolution of <0.5 mm. The use of a short TR of 5.5 milliseconds with 10 averages shortened the image acquisition from 5 minutes to a single breath hold of 17 seconds and reduced the susceptibility to motion artifacts. A higher number of pixels representing the bladder wall and high readout bandwidth of 574 Hz/pixel ensure that the mis-mapping of the water and fat signals is limited to a pixel (i.e. 0.7 pixels = 440 Hz/574 Hz) ^66, 78^. Contrast-enhanced MRI (CE-MRI) of the ulcerative IC/BPS patient after the instillation of NCM revealed segmentation into three layers of different signal intensity in post-contrast images taken at the FA of 14°, where the middle layer of bright signal intensity was sandwiched between the inner and outer layers of lower signal intensity. The constant TR of 5.5 milliseconds at different FAs achieves the stable steady-state conditions necessary for the signal intensity measurement of the same slice at different FA to become a function of the T _1_ relaxation time ( Figure 4G and 4H).

In a study published by another group, GBCA was instilled at the rate of 2–2.5 cc per second over a 5–10 minute period or a total volume of 600–800 mL ^86^. In comparison, a standard volume of 50 mL for NCM instillation is likely to be more tolerable for a subset of severe IC/BPS patients with low bladder capacity of 200–400 mL ^7, 89^. Bladder wall thinning of approximately 0.41 mm measured after 50 mL instillation for NCM-enhanced T _1_-weighted MRI ^61^ is consistent with the ultrasound measurements of BWT distended to similar volumes ^46^. It is known that overdistention of the bladder wall can provoke motion artifacts in sensitive patients and the consequent thinning of the bladder wall can also hinder the differentiation of tissue layers and the luminal texture ^90^.

**Pelvic magnetic resonance imaging as a tool to assess the contribution of bladder permeability in interstitial cystitis/bladder pain syndrome**

Instead of serum uptake, T _1_-weighted CE-MRI in IC/BPS patients relies on the penetration of an instilled GBCA into the bladder wall as a non-invasive measure of bladder permeability ^86, 91, 92^. Towner *et al*. reported that instillation of GBCA bestowed greater skewness and kurtosis in the probability distribution for the bladder wall signal intensity ^86^. It is worth stating here that the signal intensity measurement in MRI depends on several factors, including intrinsic properties of the tissue and the acquisition parameters, receiver coil geometry, sensitivity, and signal amplifier gains. These variables introduce a non-linearity into the signal intensity measurement and differences in hardware used at different places preclude any direct comparisons of intensity values across patients or imaging centers. Moreover, hypointense signal intensity in the inner layer of the bladder wall ( Figure 1C and Figure 3) after NCM instillation indicates that quantitative measurement of the bladder wall signal intensity following GBCA instillation is especially vulnerable to the MRI artifacts ^62, 66, 77, 78^.

Passive diffusion of gadobutrol into the bladder wall is presumed to create a downhill concentration gradient of GBCA with the highest concentration in the innermost layer. However, signal intensity in different tissue layers of the bladder wall does not show a similar downhill gradient owing to the T _2_ relaxation effect of GBCA at higher concentrations ^80, 83^, which reduces the relative signal intensity of the inner layer compared to the middle layer of the bladder wall. The T _2_ relaxation effect of the GBCA excreted in the urine is also known to cause pseudolayering in urine containing excreted GBCA accumulating in the bladder after intravenous injection of the GBCA ^80^. Also, a role for an increased proton dephasing from localized perturbation of the magnetic field by ferumoxytol cannot be ruled out in the lowering of the signal intensity in the inner layer. Since signal intensity measurement in CE-MRI can introduce errors in bladder wall permeability measurement, the interpretation of the imaging data can be simplified by direct calculation of the proton density and spin relaxation times. Hence, acquisition T _1_ relaxation time can facilitate improved characterization of the bladder wall, enhance image tissue contrast, and provide a more direct link between the observed signal changes and pathological changes detected by bladder wall biopsy. Besides, quantitative relaxometry (quantitative measurement of T _1 _relaxation time) can facilitate the standardization of bladder wall imaging for longitudinal study on the same patient and multicenter studies.

In a small pilot study of six subjects, we used the differences in signal intensity at different FAs to calculate the T _1_ relaxation time, which is an intrinsic property of tissues. T _1_ graphically represents the first order time constant required for the z-component of net magnetization to reach (1-1/e) or about 63% of its maximum signal intensity ^93^. A change in the longitudinal relaxation rate (1/T _1_) of the tissue is directly proportional to the GBCA concentration in the tissue ^94^. However, the acquisition of artifact-free, high-resolution imaging of human bladder wall is a prerequisite for mapping the T _1_ relaxation time and for deriving the bladder wall permeability data. Therefore, automatic acquisition of the pre-contrast and post-contrast pixel wise T _1_ maps obtained after NCM ( Figure 4G and 4H) can be a robust measure of bladder wall permeability. The variable FAs ^95^ method is a preferred alternative to the Look-Locker method for quantitative measurement of bladder wall T _1_, as it does away with the inversion pulse and clinically infeasible times required to more fully characterize the recovery curves. The differences in signal intensity at different FAs in different layers of the bladder wall were transformed to a homogenous blue color indicating the shortening of the bladder wall T _1_ following diffusion of gadobutrol from instilled NCM ( Figure 4).

Studies on the heart and liver have demonstrated that fibrotic changes ^96, 97^ increase the T _1 _relaxation time and therefore quantitative T _1_ measurement ^98^ of the bladder wall is proposed as an objective and reproducible parameter for the non-invasive detection of diffuse histological changes such as edema and fibrosis without resorting to bladder wall biopsy. Indeed, the bright middle layer in the bladder wall ( Figure 1C, Figure 3B, and Figure 4F) is consistent with the evidence of fibrosis noted in the bladder biopsy and bladder wall thickening noted in CT of the same IC/BPS patient. Therefore, quantitative measurement of bladder wall T _1_ can become a non-invasive biomarker of diffuse tissue changes leading to increased bladder wall permeability, which can help the clinician to discriminate IC/BPS from other pelvic floor defects. In recent years, the use of T _1_ mapping techniques has been simplified and can be readily integrated into clinical MRI examination.

**Pelvic magnetic resonance imaging as a tool to assess the contribution of pelvic floor hypertonicity in interstitial cystitis/bladder pain syndrome**

The anatomies of pelvic structures ^99, 100^ are critical for the diagnosis of pelvic floor hypertonicity. The contribution of pelvic floor hypertonicity to pain in 15 female IC/BPS patients and age-matched controls was investigated with T _2_-weighted MRI without instilling or injecting any contrast agents. Increased pelvic floor hypertonicity in IC/BPS patients was linked to shortened levator muscles, wider posterior puborectalis angle, and decreased puborectal distances. While the total urethral length and M line were similar in two cohorts, the H line was shorter and the vaginal cuff and bladder neck distances to the H line were longer in patients with IC/BPS. These observations need to be considered in light of the known age dependence in the displacement of the bladder and vagina ^99^ and as-yet-unknown contribution of neuronal factors, decrease in muscle strength/mass, or fat deposition. Pelvic magnetic resonance 3D reconstructed images can reveal the anatomical relationships of pelvic organs with each other ^101^.

**Brain magnetic resonance imaging as a tool to assess the contribution of central pain processing**

In a multicenter study, high-resolution T _1_-weighted MRI ^11^ and functional MRI (fMRI) ^12^ of brain was used to detect alterations in central pain processing of well-phenotyped IC/BPS patients. Compared to healthy controls, the pain, mood (anxiety), and urological symptoms of 33 IC/BPS patients were associated with a notably elevated volume of gray matter in a number of different brain regions. A separate study examined the 10-minute resting brain fMRI of 85 IC/BPS patients and 85 female healthy controls ^12^ to detect blood oxygen level-dependent signal, which was then transformed to the frequency domain. Altered frequency distributions in viscerosensory (post insula), somatosensory (postcentral gyrus), and motor regions (anterior paracentral lobule and medial and ventral supplementary motor areas) were detected in IC/BPS patients relative to controls. IC/BPS patients also showed increased functional connectivity of the anterior paracentral lobule and medial and ventral supplementary motor areas to the midbrain (red nucleus) and cerebellum. Patients who experienced pain during bladder filling had the highest level of increased functional connectivity.

## Urine analysis of interstitial cystitis/bladder pain syndrome patients

Our group reported that IL-8 (CXCL-8) is elevated, along with other members of the CXC family of chemokines namely CXCL-1 and CXCL-10, in the urine of Hunner-type IC/BPS patients ^47^. Erickson *et al*. found a positive association between elevated levels of CXCL-8 in the urine and bladder mast cell counts of IC/BPS patients ^102^. A subsequent study from her group reproduced the earlier reported elevation of CXCL-10 in Hunner-type IC/BPS patients ^39^. Longitudinal analysis of the urine samples of IC/BPS patients at baseline and at follow-up further demonstrated the treatment-associated reduction in urinary chemokine levels following hydrodistension ^103^ and sacral neuromodulation ^104^ at 4 and 24 weeks, respectively. A recent crowd-sourcing urine study confirmed the elevation of CXCL-1 and CXCL-8 in the urine of 153 IC/BPS patients ^105^. Nerve growth factor (NGF) ^106^ is overexpressed in IC/BPS patients, and a recent meta-analysis of several studies found increased NGF levels in the urine of IC/BPS patients ^107^. NGF overexpression in the bladder was linked to the deposition of type I collagen in the extracellular matrix of rat bladder ^108^. TGF-β1 is another signaling mediator shown to be responsible for fibrosis in rat bladder following exposure to ketamine ^109^ or cyclophosphamide ^35^.

## Potential applications of imaging tools in interstitial cystitis/bladder pain syndrome

The identification of patients primarily afflicted with organic disease of the bladder wall ^7^ and those with myofascial pain or disturbance in central pain processing can rationalize the clinical management of incipient IC/BPS. We envision that NCM-enhanced T _1_-weighted pelvic MRI can provide structural and functional imaging of the bladder wall to easily discriminate patients who have organic disease of the bladder wall from those who have pelvic floor hypertonicity. Only available current option of bladder wall biopsy for phenotyping IC/BPS patients with organic disease of the bladder wall, is invasive and riddled with potential complications, which makes it likely that such patients are under-represented in the numerous failed clinical trials on anti-inflammatory ^110, 111^ or GAG replacement ^112, 113^ therapies. Therefore, there is an unmet need for a non-invasive imaging method for assessing chronic inflammation in bladder wall IC/BPS, which can enable the selection of IC/BPS patients most likely to respond to anti-inflammatory therapies. IC/BPS patients with organic disease of the bladder wall are expected to demonstrate higher bladder wall permeability for GBCAs, and such patients will be good candidates for GAG replacement therapy by pentosan polysulfate or other new intravesical treatments including liposomes, submucosal injection of steroids into the bladder wall ^114^, or Vessilen ^®^ (a new formulation of 2% adelmidrol [the diethanolamide derivative of azelaic acid] + 0.1% sodium hyaluronate) ^115^. On the other hand, IC/BPS patients who do not exhibit bladder permeability changes on MRI are more likely to benefit from therapies directed at pelvic floor or central disturbances.

Fibrotic changes (collagen deposition) in the bladder wall are a well-known outcome of progressive IC/BPS ^15, 33, 34, 45^, ketamine abuse ^41, 116^, and obstruction ^117, 118^. The inability to non-invasively measure fibrosis represents a major gap in the care and investigation of IC/BPS and other voiding disorders. Large volumetric data available from MRI can allow reconstruction of the bladder wall and pelvic structures ^101^. Hunner’s lesions and fibrotic changes in the bladder wall can be visualized using multiphase data sets acquired in a continuous fashion using novel free-breathing MRI sequence without predetermined temporal resolution, allowing for retrospective sparse reconstruction at flexible temporal resolution ^119^. Increased permeability of the bladder wall can also be confirmed by the penetration and accumulation of 2-deoxy-2-[fluorine-18] fluoro-D-glucose in the bladder wall using positron emission tomography integrated with CT ( ^18^F-FDG PET/CT). PET with ^18^F-FDG, an analogue of glucose, provides valuable functional information based on the increased glucose uptake in the inflamed sites in the bladder wall before morphological alterations occur. The combined acquisition of PET and CT has synergistic advantages over PET or CT alone, as the combined approach minimizes individual limitations of each technique. Imaging tools have the potential to replace the subjective impressions of patients in objectively quantifying symptoms and the clinical response of new drugs. The weak correlation between symptom questionnaire scores and patient satisfaction is considered a potential bottleneck in the continued scientific progress and new drug development for IC/BPS.

## Conclusions

Recent advances in MRI of the bladder wall and brain can transform our understanding and care of patients with IC/BPS. High-resolution T _1_-weighted MRI and fMRI can inform on the disturbances in central processing of chronic pelvic pain. CE-MRI of the bladder wall has the potential to objectively separate three distinct entities of IC/BPS: pelvic hypersensitivity, IC/BPS, and IC/BPS with Hunner's lesions. Such objective classification can enable proper patient selection for drugs targeting chronic inflammation in the bladder wall and reduce the reliance on subjective outcomes for predicting the efficacy and safety of novel therapeutic interventions.

## Abbreviations

BWT, bladder wall thickness; CE-MRI, contrast-enhanced magnetic resonance imaging; CNR, contrast–noise ratio; 2D, two dimensional; 3D, three dimensional; ESSIC, European Society for the Study of Interstitial Cystitis/bladder pain syndrome; FA, flip angle; FLASH, fast low angle shot; fMRI, functional magnetic resonance imaging; FOV, field of view; GAG, glycosaminoglycan; GBCA, gadolinium-based contrast agent; IC/BPS, interstitial cystitis/bladder pain syndrome; ICS, International Continence Society; MRI, magnetic resonance imaging; NGF, nerve growth factor; NIR, near infra-red; NIRS, near infra-red spectroscopy; PET, positron emission tomography; SNR, signal-to-noise ratio; SPIO, superparamagnetic iron oxide; T, Tesla; TE, echo time; TGFβ1, transforming growth factor β1; TR, repetition time, UTI, urinary tract infection
